# Time-Restricted Feeding Protects against Renal Ischemia-Reperfusion Injury in Mice

**DOI:** 10.3390/ijms25147652

**Published:** 2024-07-12

**Authors:** Do Kyun Kim, Young Suk Kim, Min Jeong Kim, Seo Rin Kim, Dong Won Lee, Soo Bong Lee, Il Young Kim

**Affiliations:** 1 Department of Internal Medicine, Pusan National University School of Medicine, Yangsan 50612, Republic of Korea; evencoast@naver.com (D.K.K.); seorink96@gmail.com (S.R.K.);; 2 Research Institute for Convergence of Biomedical Science and Technology, Pusan National University Yangsan Hospital, Yangsan 50612, Republic of Korea

**Keywords:** acute kidney injury, ischemia-reperfusion injury, time-restricted feeding

## Abstract

Ischemia-reperfusion injury (IRI) in the kidneys is a major cause of acute kidney injury (AKI). Time-restricted feeding (TRF), known for its metabolic health benefits and alleviation of various chronic diseases without calorie restriction, was investigated for its potential protective effects against IRI-induced AKI. Male C57BL/6 mice underwent unilateral IRI, with their kidneys collected after two days. For two weeks before IRI induction, the TRF group had unlimited access to standard chow but within an 8-hour feeding window during the dark cycle. The study groups were Control, TRF, IRI, and TRF + IRI. In the TRF + IRI group, tubular damage scores significantly decreased compared to the IRI group. Furthermore, the TRF + IRI mice had lower levels of phosphorylated NF-κB and fewer F4/80-positive macrophages than the IRI group. Oxidative stress markers for lipids and proteins were also notably lower in the TRF + IRI group. Additionally, TUNEL-positive tubular cells and cleaved caspase-3 expression were reduced in the TRF + IRI group. Without calorie restriction, TRF mitigated renal damage by reducing inflammation, oxidative stress, and tubular apoptosis in renal IRI. This suggests that TRF could be a promising dietary strategy to prevent IRI-induced AKI.

## 1. Introduction

Acute kidney injury (AKI) is characterized by a sudden decline in kidney function, representing a major worldwide health issue due to its increased morbidity and mortality rates [1,2]. Renal ischemia-reperfusion injury (IRI) stands as a primary instigator of AKI and manifests in clinical scenarios including cardiovascular surgery, kidney transplantation, and instances of hemorrhagic, traumatic, and septic shock [3]. Despite substantial strides in understanding the pathophysiology of IRI-induced AKI over recent decades, effective prophylactic measures remain elusive. Hence, the pursuit of novel prophylactic strategies for IRI-induced AKI holds paramount clinical importance.

Calorie restriction, which involves reducing caloric intake without inducing malnutrition, consistently demonstrates efficacy in reducing body weight and extending healthy lifespans across diverse species [4]. Moreover, it promotes metabolic well-being and mitigates chronic metabolic ailments such as type 2 diabetes and cardiovascular disorders [5]. In the realm of kidney disease, calorie restriction stimulates an adaptive defense mechanism, enhancing stress resistance, and mitigating renal IRI in experimental animal models [6]. Consequently, endeavors have been initiated to investigate an alternative, dietary-focused intervention aimed at reducing organ injury in both animal models and human subjects. However, despite numerous investigations underscoring the favorable effect of calorie restriction in humans, findings from obesity intervention trials in recent decades indicate significant challenges in sustaining daily calorie restriction over extended periods for the vast majority of individuals [4].

Time-restricted feeding (TRF), a novel dietary strategy allowing animals access to food within specific daily time intervals, emerges as a compelling alternative to calorie restriction, showing promise in delivering comparable benefits for weight management and cardiometabolic health [7]. Notably, TRF eliminates the need for calorie counting by not restricting caloric intake during the designated feeding window, thereby enhancing adherence [8]. Although preclinical and clinical trials have underscored TRF’s beneficial effects on a range of conditions, such as cancers, diabetes, cardiovascular disease, obesity, neurodegenerative disorders, asthma, and arthritis [7], its potential impact on renal health has received limited attention. The aim of this study is to investigate whether TRF offers protection against IRI-induced AKI in a mouse model.

## 2. Results

### 2.1. TRF Mitigates Pathological Damage in IRI-Induced AKI

**Figure 1:**
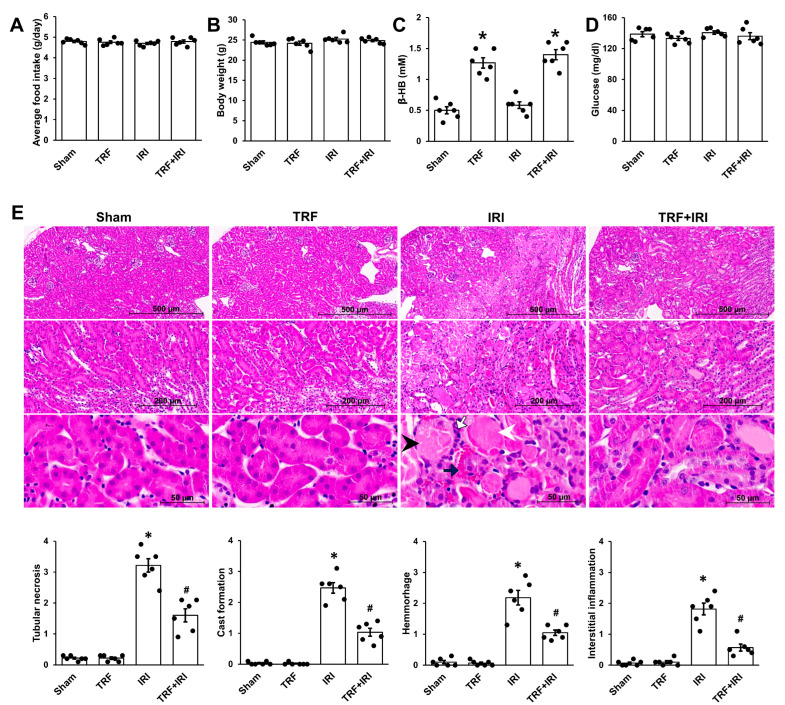
TRF’s impact on kidney damage in renal IRI. (**A**) Food intake. (**B**) Body weight. (**C**) β-HB level. (**D**) Glucose level. (**E**) Exemplary images of H&E staining. Tubular necrosis (black arrowhead), cast formation (white arrowhead), hemorrhage (black arrow), and interstitial inflammation (white arrow). The results are shown as means ± standard deviations. There were six subjects per group. * *p* < 0.05 compared to the sham group. # *p* < 0.05 compared to the IRI group.

For a period of two weeks, mice subjected to TRF consumed a comparable amount of food to the Control group fed ad libitum, confirming the absence of caloric restriction during TRF (Figure 1A). Notably, TRF did not result in any reduction in body weight (Figure 1B). Moreover, mice subjected to TRF exhibited elevated levels of β-HB, indicating the induction of ketosis by the TRF regimen (Figure 1C). There was no discernible variance in blood glucose levels between mice subjected to TRF and those fed ad libitum (Figure 1D).

Subsequently, histological evaluations were conducted on kidney sections stained with H&E for each experimental group (Figure 1E). The IRI group exhibited significant renal damage compared to both the sham-operated and TRF groups. The most significant damages were localized in the outer medulla, exhibiting tubular necrosis, hemorrhage, cast formation, and interstitial inflammation. However, these injuries were markedly alleviated in the TRF + IRI group.

### 2.2. TRF Mitigates Inflammation in IRI-Induced AKI

**Figure 2:**
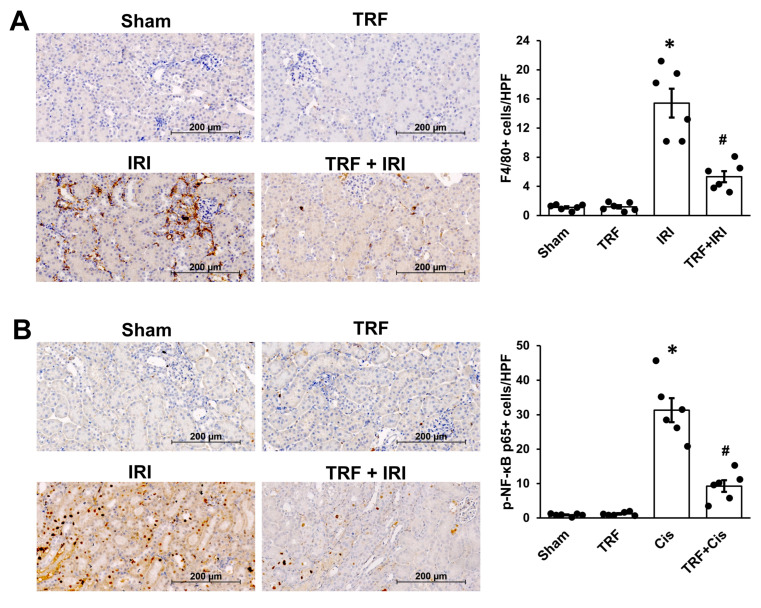
TRF’s impact on inflammation in renal IRI. Exemplary images of immunohistochemical analyses for F4/80 (**A**) and p-NF-κB p65 (**B**). The results are shown as means ± standard deviations. There were six subjects per group. * *p* < 0.05 compared to both the sham and TRF groups. # *p* < 0.05 compared to the IRI group.

In our investigation of the molecular mechanisms underlying IRI-induced AKI, we focused on F4/80, which indicates macrophage infiltration within kidney tissues (Figure 2A). We noticed an increased number of F4/80-stained cells, signifying macrophage infiltration, in the interstitial space of the IRI group, while the TRF + IRI group showed a significant reduction in the count of F4/80-stained cells relative to the IRI group.

Recognizing NF-κB’s pivotal role in modulating the inflammatory process [3,9], we examined NF-κB expression dynamics in response to inflammation induced by IRI in AKI (Figure 2B). Immunohistochemical analyses revealed a heightened expression of p-NF-κB p65 in the IRI mice, whereas the sham mice exhibited lower levels. However, notably, the expression of p-NF-κB p65 was significantly diminished in the TRF + IRI group compared to the IRI group, highlighting the potential anti-inflammatory impact of TRF in mitigating inflammation associated with IRI.

### 2.3. TRF Mitigates Oxidative Stress in IRI-Induced AKI

**Figure 3:**
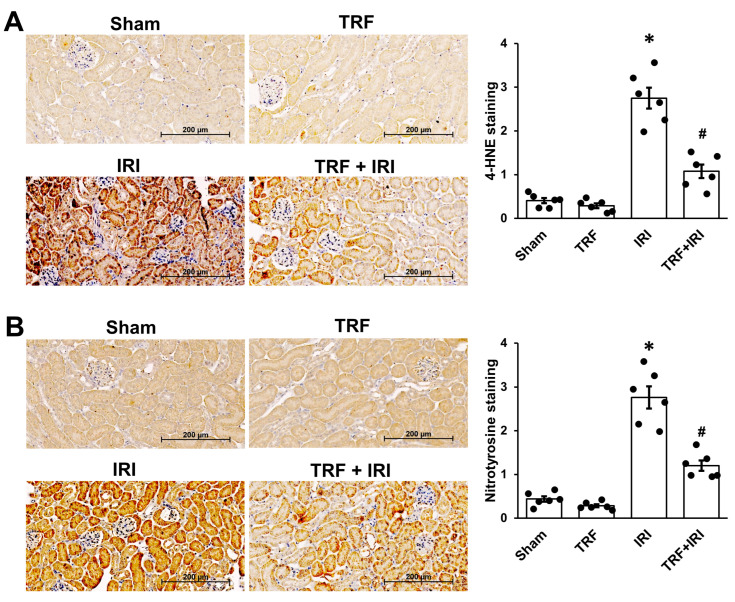
TRF’s impact on oxidative stress in renal IRI. Exemplary images of immunohistochemistry for 4-HNE (**A**) and nitrotyrosine (**B**). The results are shown as means ± standard deviations. There were six subjects per group. * *p* < 0.05 compared to both the sham and TRF groups. # *p* < 0.05 compared to the IRI group.

Acknowledged as a substantial factor in renal IRI [9], oxidative stress prompted an inquiry into TRF’s capacity to alleviate renal IRI. Kidney sections underwent antibody staining targeting 4-HNE to evaluate lipid oxidation (Figure 3A) and nitrotyrosine for protein oxidation (Figure 3B) to assess this potential. Our findings revealed a notable elevation in the expression levels of nitrotyrosine and 4-HNE in the IRI group compared to the sham group. However, TRF significantly ameliorated these alterations, suggesting its potential in mitigating oxidative stress associated with IRI-induced AKI.

### 2.4. TRF Mitigates Tubular Apoptosis in IRI-Induced AKI

**Figure 4:**
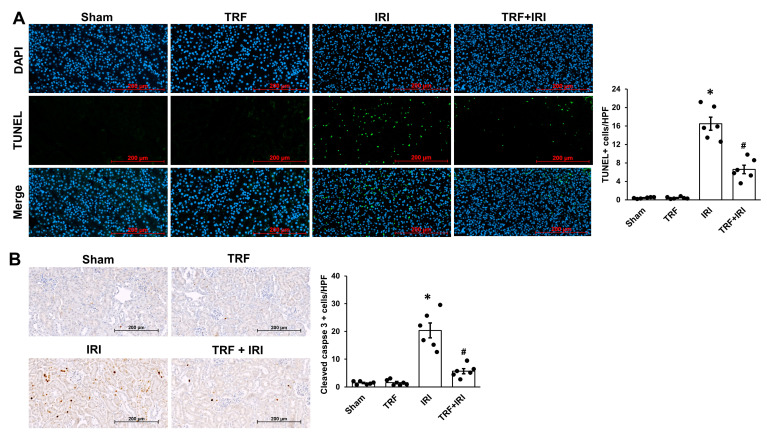
TRF’s impact on tubular apoptosis in renal IRI. Exemplary image of TUNEL staining (**A**) and immunohistochemical analyses for cleaved caspase-3 (**B**). The results are shown as means ± standard deviations. There were six subjects per group. * *p* < 0.05 compared to both the sham and TRF groups. # *p* < 0.05 compared to the IRI group.

Besides tubular necrosis, tubular apoptosis holds significant importance in IRI-induced AKI [9]. Therefore, we investigated whether TRF could alleviate tubular apoptosis in this context. TUNEL staining was utilized to assess tubular cell apoptosis. The IRI group exhibited markedly higher levels of tubular apoptosis than the sham group (Figure 4A). In contrast, the TRF + IRI group demonstrated a reduction in tubular apoptosis relative to the IRI group.

Immunohistochemical assessments were carried out to probe caspase-3, the predominant caspase involved in cellular apoptosis. The intensity of staining for active (cleaved) caspase-3, indicating its activation, was markedly elevated in the IRI group compared to the sham group (Figure 4B). In contrast, the TRF + IRI mice exhibited a significant reduction in the presence of activated caspase-3 relative to the IRI mice.

## 3. Discussion

The pathophysiology of IRI-induced AKI is intricate, with specific mechanisms still not fully understood [3,9]. Nonetheless, mounting evidence indicates that inflammatory reactions, oxidative stress, and tubular cell apoptosis are pivotal factors [3]. Among these factors, the initiation of a robust inflammatory response by IRI is pivotal in both functional and structural kidney deterioration [3]. Renal IRI triggers an inflammatory cascade, exacerbating renal damage, thus targeting inflammation emerges as a crucial therapeutic approach to preserve renal tissue [9]. Additionally, oxidative stress contributes to the pathogenesis of renal IRI. The increased production of reactive oxygen species (ROS) following IRI activates various signaling pathways, leading to damage and the death of renal tubular epithelial cells [9]. Consequently, addressing oxidative stress represents a promising strategy for tissue protection during IRI. Moreover, substantial emphasis has been placed on the apoptosis of tubular epithelial cells as a critical component of the mechanisms driving renal IRI [9]. Several investigations have suggested that suppressing tubular cell apoptosis mitigates the impact of renal IRI [3,9]. In the context of these established mechanisms, the present study emphasizes the protective effects of TRF against renal IRI, attained by quelling inflammation, oxidative stress, and tubular apoptosis.

While previous studies have suggested the protective effects of calorie restriction or fasting against renal IRI, there has been limited research on the specific benefits of TRF for IRI-induced AKI. Rojas-Morales et al. demonstrated that short-term TRF provided protec-tion against renal IRI in a rat model [5]. Most studies on TRF’s beneficial effects on health and disease involve a restricted feeding period of 6 to 10 h per day. However, extreme conditions, such as the 2 h per day restricted diet used in the study by Rojas-Morales et al., may maximize TRF effects in a short time frame but are impractical for human application. In contrast, our TRF protocol, which allows an 8-hour window for food intake, is more practical for real-life implementation. Indeed, the TRF protocols currently recommended for humans also advocate for a feeding period of 6 to 10 h per day [7]. Therefore, although our study is not the first to explore TRF’s effects on IRI-induced AKI, it adds to the growing body of evidence supporting TRF’s benefits by demonstrating that an 8-hour TRF, which is highly feasible, is effective in mitigating IRI-induced AKI.

Numerous studies have delved into the advantages of TRF concerning health and disease [7]. While some attribute TRF’s positive outcomes to a decrease in overall food consumption, others have shown enhancements in cardiometabolic indicators and defense against pathological conditions, even with the application of isocaloric diets [10]. In our investigation, mice in the TRF group displayed akin body weights and ingested comparable quantities of food as the sham group, indicating an absence of calorie restriction. This implies that the beneficial impacts of TRF on IRI-induced AKI in our study did not stem from decreased caloric intake.

One pivotal finding in our study is the notable attenuation of inflammation by TRF in renal IRI. Although the exact mechanisms underlying the anti-inflammatory effect of TRF on renal IRI were not fully elucidated in the present investigation, TRF is renowned for its inherent anti-inflammatory properties. Previous studies, encompassing both human and animal research, have underscored TRF’s ability to dampen inflammatory responses across a spectrum of chronic inflammatory conditions, spanning from cardiovascular disease and diabetes to neurodegenerative disorders and arthritis [7,11]. In the realm of kidney disease, TRF has been shown to diminish renal innate immune cells in a mouse model of hypertension [10]. Despite these intriguing observations, the exact mechanism by which TRF mitigates inflammation in our study remains enigmatic. One plausible avenue is TRF-induced ketosis. In TRF-treated mice, we observed elevated levels of β-HB, suggesting the induction of a ketotic state by the TRF regimen. As a byproduct of hepatic fat breakdown, β-HB functions as an alternative energy source in the absence of carbohydrates [12]. Besides its metabolic role, β-HB emerges as a pivotal signaling molecule, possessing a distinct capacity to regulate inflammatory reactions [12]. We contend that further investigation is crucial to unravel the intricate mechanisms behind TRF’s anti-inflammatory effects in the context of renal IRI.

Our investigation has unveiled TRF’s ability to suppress oxidative stress in a mouse model of IRI-induced AKI. Although our study did not fully elucidate TRF’s mechanism in countering renal IRI-induced oxidative stress, there is mounting evidence suggesting TRF’s antioxidative properties. Previous research in both animals and humans has hinted at TRF’s potential to lower oxidative stress markers in chronic diseases [11]. While the molecular pathways remain unclear, it is conceivable that redox-sensitive transcriptional regulators might be involved in mediating TRF’s beneficial effects on oxidative stress [5]. Furthermore, β-HB induced by TRF has been observed to stimulate various transcription factors, thus regulating cytoprotective genes associated with ROS [13]. Future investigations are essential to uncover the complex mechanism underlying TRF’s antioxidative impact on renal IRI.

The present study also emphasized TRF’s capability to attenuate tubular apoptosis in renal IRI. Our results echo prior studies that underscore TRF’s capacity to prevent cell death across diverse animal models. Notably, TRF has demonstrated efficacy in mitigating apoptosis in a mouse model of hepatic IRI [14] and in reducing apoptotic events through autophagy modulation in rats with doxorubicin-induced cardiotoxicity [15]. In the domain of kidney ailments, preoperative TRF administration has shown promise in shielding against renal IRI by curbing apoptosis in a mouse model of aortic aneurysm [16]. Despite these promising discoveries, the exact mechanism by which TRF reduces tubular apoptosis in renal IRI remains elusive. Hence, further investigations are warranted to unravel TRF’s impact on tubular apoptosis within the realm of renal IRI.

## 4. Materials and Methods

### 4.1. Animals

Male C57BL/6 mice (8 weeks old) were housed in a specific pathogen-free facility. To induce renal IRI, unilateral IRI was performed without contralateral nephrectomy by clamping the right renal pedicle for 30 min, as previously described [17]. The mice were allocated into four groups, each consisting of six individuals: (a) sham: provided ad libitum food without renal IRI, (b) TRF: subjected to TRF without renal IRI, (c) IRI: provided ad libitum food and subjected to renal IRI, and (d) TRF + IRI: subjected to TRF and renal IRI. Mice with ad libitum access had continuous access to food throughout the day, whereas mice on TRF were provided with food for 8 h daily, commencing 3 h after the onset of the dark cycle (from 9:00 p.m. to 5:00 a.m.), for a total of 2 weeks. Following 2 weeks of either ad libitum feeding or TRF, unilateral IRI was induced in the IRI group and the TRF + IRI group. Mice in both the sham and TRF groups underwent the same surgical protocols, with the exception of renal pedicle manipulation. The mice were euthanized 48 h after renal IRI.

### 4.2. Body Weight, food Intake, and Blood Measurements

Blood glucose and β-hydroxybutyrate (β-HB) levels were assessed using a device (FreeStyle Optium Neo, Abbott, North Chicago, IL, USA) in whole blood collected from the tail vein of mice after 2 weeks of either TRF or ad libitum feeding, just before renal IRI. Body weight and food intake were also measured before renal IRI, concurrently with blood collection.

### 4.3. Histology

The kidney samples, preserved in paraffin, were sectioned into 4 μm slices and underwent staining with hematoxylin and eosin (H&E) using established protocols [17,18]. Subsequently, all stained kidney tissues were digitally scanned using a ZEISS Axioscan7 microscope (Carl Zeiss, Jena, Germany). Images were captured at 20× magnification utilizing ZEN Lite microscope software (Carl Zeiss, version 3.9), and ten high-power fields (HPFs) were randomly selected for analysis. Histopathological alterations in the cortex and medulla were evaluated by a pathologist in a blinded manner, with a focus on parameters such as tubular necrosis, hemorrhage, cast formation, and interstitial inflammation. The severity of these changes was graded using the following scale: Score 0, less than 10%; Score 1, 10–25%; Score 2, 25–50%; Score 3, 50–75%; and Score 4, 75–100% [18].

### 4.4. Immunohistochemistry

Immunohistochemistry assessments were carried out on 4 μm paraffin-embedded kidney slices using established protocols [17,18]. Primary antibodies used were anti-F4/80 (#ab111101, Abcam, Cambridge, UK), anti-phosphorylated nuclear factor-κB (p-NF-κB) p65 (#sc-136548, Santa Cruz, CA, USA), anti-cleaved caspase-3 (#9961, Cell Signaling Technology, Danvers, MA, USA), anti-4-hydroxy-2-nonenal (4-HNE) (JalCA, Sizuoka, Japan), and anti-nitrotyrosine (#ab125106, Abcam, Cambridge, UK). Following the imaging of stained kidney sections, ten HPFs were randomly selected for assessment. F4/80- and p-NF-κB p65-positive cell counts per HPF were assessed in a blinded fashion. Staining for 4-HNE and nitrotyrosine was graded semi-quantitatively on a scale from 0 to 4, indicating the percentage of stained area: 0 for 0%, 1 for 1–25%, 2 for 26–50%, 3 for 51–75%, and 4 for >75% [17,18].

### 4.5. Terminal Deoxynucleotidyl Transferase (TdT)-Mediated Deoxyuridine Triphosphate Nick End Labeling (TUNEL) Staining

Tubular apoptosis was evaluated using the TUNEL assay, following standardized protocols [18]. The TUNEL staining was conducted with the TUNEL Apoptosis Detection Kit (#G3250, Promega, Madison, WI, USA). After capturing images of the stained kidney tissues, TUNEL-positive cell counts were determined in ten HPFs and averaged by an observer blinded to the samples.

### 4.6. Statistical Analysis

The results were presented as mean ± standard deviation (SD). Statistical analyses were performed using appropriate methods, including the Kruskal–Wallis test or Mann–Whitney U test, based on data distribution. All statistical computations were conducted with SPSS 26.0 (SPSS Inc., Chicago, IL, USA). A significance threshold of *p* < 0.05 was utilized to establish statistical significance.

## 5. Conclusions

Our study unveiled escalated inflammatory responses, oxidative stress, and tubular apoptosis in AKI triggered by IRI. TRF has emerged as a promising intervention, alleviating renal IRI by reducing inflammatory reaction, oxidative stress, and tubular cell apoptosis. Therefore, TRF represents a potential novel dietary approach for preventing IRI-induced AKI. We advocate for further research to unravel the underlying mechanisms driving the beneficial effects of TRF on inflammation, apoptosis, and oxidative stress.

## Data Availability

The datasets used and/or analyzed during the current study are available from the corresponding author on reasonable request.

## References

[1] Susantitaphong P., Cruz D.N., Cerda J., Abulfaraj M., Alqahtani F., Koulouridis I., Jaber B.L. (2013). Acute Kidney Injury Advisory Group of the American Society of, Nephrology World Incidence of Aki: A Meta-Analysis. Clin. J. Am. Soc. Nephrol..

[2] Sun L., Hua R.X., Wu Y., Zou L.X. (2023). Acute Kidney Injury in Hospitalized Adults with Chronic Kidney Disease: Comparing Crock, Kdigo, and Combined Criteria. Kidney Res. Clin. Pract..

[3] Kusch A., Hoff U., Bubalo G., Zhu Y., Fechner M., Schmidt-Ullrich R., Marko L., Muller D.N., Schmidt-Ott K.M., Gurgen D. (2013). Novel Signalling Mechanisms and Targets in Renal Ischaemia and Reperfusion Injury. Acta Physiol..

[4] Anton S.D., Moehl K., Donahoo W.T., Marosi K., Lee S.A., Mainous A.G., Leeuwenburgh C., Mattson M.P. (2018). Flipping the Metabolic Switch: Understanding and Applying the Health Benefits of Fasting. Obesity.

[5] Rojas-Morales P., Leon-Contreras J.C., Granados-Pineda J., Hernandez-Pando R., Gonzaga G., Sanchez-Lozada L.G., Osorio-Alonso H., Pedraza-Chaverri J., Tapia E. (2020). Protection against Renal Ischemia and Reperfusion Injury by Short-Term Time-Restricted Feeding Involves the Mitochondrial Unfolded Protein Response. Free Radic. Biol. Med..

[6] Rojas-Morales P., Leon-Contreras J.C., Aparicio-Trejo O.E., Reyes-Ocampo J.G., Medina-Campos O.N., Jimenez-Osorio A.S., Gonzalez-Reyes S., Marquina-Castillo B., Hernandez-Pando R., Barrera-Oviedo D. (2019). Fasting Reduces Oxidative Stress, Mitochondrial Dysfunction and Fibrosis Induced by Renal Ischemia-Reperfusion Injury. Free Radic. Biol. Med..

[7] de Cabo R., Mattson M.P. (2019). Effects of Intermittent Fasting on Health, Aging, and Disease. N. Engl. J. Med..

[8] Bjerre N., Holm L., Quist J.S., Faerch K., Hempler N.F. (2021). Watching, Keeping and Squeezing Time to Lose Weight: Implications of Time-Restricted Eating in Daily Life. Appetite.

[9] Han S.J., Lee H.T. (2019). Mechanisms and Therapeutic Targets of Ischemic Acute Kidney Injury. Kidney Res. Clin. Pract..

[10] Sims B.M., Goodlett B.L., Allbee M.L., Pickup E.J., Chiasson V.L., Arenaz C.M., Henley M.R., Navaneethabalakrishnan S., Mitchell B.M. (2022). Time Restricted Feeding Decreases Renal Innate Immune Cells and Blood Pressure in Hypertensive Mice. J. Hypertens..

[11] Mattson M.P., Allison D.B., Fontana L., Harvie M., Longo V.D., Malaisse W.J., Mosley M., Notterpek L., Ravussin E., Scheer F.A. (2014). Meal Frequency and Timing in Health and Disease. Proc. Natl. Acad. Sci. USA.

[12] Puchalska P., Crawford P.A. (2021). Metabolic and Signaling Roles of Ketone Bodies in Health and Disease. Annu. Rev. Nutr..

[13] Gomora-Garcia J.C., Montiel T., Huttenrauch M., Salcido-Gomez A., Garcia-Velazquez L., Ramiro-Cortes Y., Gomora J.C., Castro-Obregon S., Massieu L. (2023). Effect of the Ketone Body, D-Beta-Hydroxybutyrate, on Sirtuin2-Mediated Regulation of Mitochondrial Quality Control and the Autophagy-Lysosomal Pathway. Cells.

[14] Ren J., Hu D., Mao Y., Yang H., Liao W., Xu W., Ge P., Zhang H., Sang X., Lu X. (2019). Alteration in Gut Microbiota Caused by Time-Restricted Feeding Alleviate Hepatic Ischaemia Reperfusion Injury in Mice. J. Cell. Mol. Med..

[15] Abas E., Sabry M.M. (2020). Intermittent Fasting Attenuates Apoptosis, Modulates Autophagy and Preserves Telocytes in Doxorubicin Induced Cardiotoxicity in Albino Rats: A Histological Study. Egypt. J. Histol..

[16] Saat T.C., van der Pluijm I., Ridwan Y., van Damme-van den Engel S., van Heijningen P.M., Clahsen-van Groningen M.C., Verhagen H.J.M., IJzermans J.N.M., Essers J., de Bruin R.W.F. (2020). Pre-Operative Fasting Provides Long Term Protection against Chronic Renal Damage Induced by Ischaemia Reperfusion Injury in Wild Type and Aneurysm Prone Fibulin-4 Mice. Eur. J. Vasc. Endovasc. Surg..

[17] Kim I.Y., Park Y.K., Song S.H., Seong E.Y., Lee D.W., Bae S.S., Lee S.B. (2021). Role of Akt1 in Renal Fibrosis and Tubular Dedifferentiation During the Progression of Acute Kidney Injury to Chronic Kidney Disease. Korean J. Intern. Med..

[18] Kim M.J., Kim Y.S., Kim S.R., Lee D.W., Lee S.B., Kim I.Y. (2024). Pre-Treatment with Beta-Hydroxybutyrate Mitigates Cisplatin-Induced Acute Kidney Injury. Biochem. Biophys. Res. Commun..

